# Tales from the Jazz ASH: highlights from the 2013 American Society of Haematology meeting

**DOI:** 10.3332/ecancer.2014.390

**Published:** 2014-01-24

**Authors:** Luca Mazzarella

**Affiliations:** European Institute of Oncology, Milan 20141, Italy

**Keywords:** myeloid malignancies, lymphoid malignancies, targeted therapy, drug discovery, tyrosine kinase inhibitors, chimeric antigen receptors

## Abstract

The 55th annual ASH meeting was held in pleasant New Orleans and was the largest in its history, with 22,495 participants coming from 113 nations. A ‘bench-to-bedside and back’ attitude characterises haematology probably more than any other discipline in medicine and, as usual, this was reflected in the extremely wide breadth of the topics covered, including the last results from clinical trials and cutting-edge advancements in basic science. This year, the balance was arguably skewed: few truly clinical practice-changing results were presented. On the other hand, a great number of basic and translational studies significantly increased our understanding of the biology of numerous malignancies and heralded the coming of age of disruptive technologies. Namely, above all, next generation sequencing and T cell engineering-based cell therapy.

## Topics of general interest

### Genomics and other ‘omics’ technologies

This year’s presidential symposium was dedicated to clinical genomics, with two of the three talks on haematological malignancies.

Prof. James Downing from St Jude’s Hospital provided an overview of the Pediatric Cancer Genome Project (PCGP), a 65 million joint effort of St Jude’s and the Washington University in St Louis to decode the genomes of more than 600 paediatric cancers by sequencing matched pathological and normal samples using next generation sequencing (NGS). His talk focused on paediatric acute lymphocytic leukaemia (ALL), in his words one of the success stories of modern oncology in which cure rate has dramatically improved thanks to three main factors: the introduction of polichemotherapy, the improvement in support therapy, and the realisation that risk stratification based on underlying genetics is key to assigning the most appropriate treatment. The PCGP has dramatically increased our knowledge of the genetic alterations at the basis of paediatric leukaemias, identifying new druggable pathways. For instance, in hypodiploid ALL, the analysis identified two clearly distinct subgroups: the near-haploid type, dominated by activated RAS and other tyrosine kinases (>70% of the cases), and the low-hypodiploid type, with frequent alterations in tumour-suppressor transcription factors, such as *TP53* (91.2%), *IKZF2* (52.9%), or *RB1* (41.2%) [1]. A further significant advancement was the identification of an inversion of chromosome 16 (Inv(16)(p13.3q24.3)) in a particularly aggressive subgroup of paediatric acute megakaryoblastic leukaemias (AML). This chromosomal rearrangement leads to a chimeric CBFA2T3-GLIS2 protein which activates BMP signalling and increases haematopoietic self-renewal in experimental models [2].

Dr Matthew J Walter from Washington University in St Louis illustrated his group’s studies in elucidating the clonal evolution of secondary AMLs (sAML) in the context of myelodysplastic syndromes (MDS). Their analysis considers an extra dimension obtainable from NGS data: the variant allele frequency (VAF), obtained by dividing the number of mutant reads by the number of total reads at each locus. Mutations occur with discrete rather than continuous VAFs, so that it is possible to reconstruct the frequency of individual clones in the cell population analysed. They sequenced matched pairs of an MDS sample and its subsequent transformation into sAML. They showed that transformation is always characterised by an expansion of a founding clone already present in the MDS stage (which often contains TP53 mutations), accompanied by the sprouting of subclones bearing at least one extra coding mutation in addition to the founding one. Mutations in MDS and sAMLs tend to occur in six functional clusters: TP53, spliceosome, epigenetic modifiers, cohesin, transcription factors, and signalling transducers. Each clone almost never contains more than one mutation within the same cluster [3]. This oligoclonal architecture has important implications in the design of new targeted treatments: it suggests that only therapies aimed at mutations occurring in founding clones (like TP53) have a true chance of eradicating the disease, whereas those targeting subclonal mutations (like tyrosine kinases) are likely to provide temporary benefit by eliminating the subclone, but will inevitably fail in the long term [4].

Further along this line, a paradigm-shifting talk was offered by Dr Terrence Wong from Washington University in St Louis, who investigated the genetic basis of therapy-related AMLs. It has long been speculated that exposure to cytotoxic therapy for a previous neoplasm favours secondary leukaemias by generating genotoxic stress that leads to an increased mutation rate. This model would predict that therapy-related AMLs bear a higher number of mutations than *de novo* AML. To their surprise, after sequencing 22 cases of tAML and comparing them to data from The Cancer Genome Atlas (TCGA), the authors found no evidence of such increased number. However, by looking for now known TP53 mutations at ultra-high sequencing depth in bone marrow samples banked 3–7 years earlier, they demonstrated that a mutated clone was already present at extremely low levels prior to the development of overt AML. As mutated p53 confers resistance to chemotherapy, the authors inferred that treatment acts by *selecting*, rather than *inducing*, pre-existing TP53-mutated clones that arise as an effect of normal aging.

Advancements in ‘omics’ technologies were not limited to nucleic acid sequencing. Wendy Fantl from Garry Nolan’s laboratory in Stanford presented their results using the recently developed mass cytometry technology. In mass cytometry, cells are stained with isotope-labelled antibodies and analysed through a mass spectrometer. This allows the characterisation of up to 50 markers at the same time, overcoming the limitations of standard fluorescence cytometry. Thus they obtained a data-dense picture of normal and malignant haematopoiesis that integrates phenotypic and dynamic parameters, such as drug response or signalling dynamics. Among their most notable results in ALL, most striking was the finding that mutation-containing clones invariably differentiate to some extent following paths analogous to normal haematopoiesis. This demonstrates that the real abnormality in leukaemia lies in the proportion of cells residing in each developmental stage, not in a completely askew differentiation pathway. Importantly, each developmental stage responded in widely different manner to drugs, suggesting new avenues for identifying mechanisms of drug resistance.

### Chimeric antigen receptors

A special interest session this year was devoted to the topic of chimeric antigen receptor (CAR)-based therapy, which promises to truly revolutionise the treatment of relapsing-refractory malignancies. The method, pioneered by Carl June and colleagues in Philadelphia, consists in transducing autologous T cells with vectors encoding synthetic T cell receptor (TCR)-resembling molecules that confer antitumour specificity to the transduced lymphocyte. The synthetic receptor consists of an antigen-targeting extracellular portion coupled with a signal-transducing intracellular arm. The engineered T cells are expanded *in vitro* and introduced intravenously in the patient, where they proliferate and selectively attack the antigen-expressing tumour cells [5].

Groups from the MSKCC (Davila *et al*), the University of Pennsylvania (Grupp *et al*, Kalos *et al*, and Porter *et al*, all from Carl June’s group), the NCI (Dr Lee and Dr Kochenferder from Steve Rosenberg’s laboratory) presented data from pilot or phase I trials with anti-CD19 CAR-engineered T cells in paediatric and adult relapsed/refractory B cell malignancies (CLL, ALL, and non-Hodgkin lymphomas, and in one case a CD19-expressing T cell leukaemia). The results are extremely encouraging, with response rates between 60% and 80%, especially considering that all patients were heavily pretreated. Engineered T cells persisted for 1–15 months after infusion, resulting in complete B cell aplasia but without significant infections. Graft versus host disease (GvHD) had an extremely low incidence in the order of 10%.

The most common toxicities were infusional fevers, developingwithin the initial 24 h and only lasting for up to few days, and two mechanistically distinct delayed responses: cytokine release syndrome (CRS), characterised by dramatic (>1000×) IL6 and IFN*γ* elevation and in some cases respiratory and haemodynamic instability, and macrophage activation syndrome (MAS) with very high levels of ferritin and coagulopathy. These were not correlated with the infused cell dose but were associated with better responses; however, all were manageable with corticosteroids and the IL6-receptor antagonist tocilizumab.

Dr Ramos *et al* from the Baylor College ofMedicine reported on the only study in which CARs were designed against a target other than CD19, the κ-light chain. By exploiting the clonality of light chain expression on malignant cells, this strategy should ideally avoid the pan-B cytopenia observed with CD19-targeting CARs. Treated patients included CLL, myeloma, and NHL. The approach was shown to be feasible and although the numbers were still relatively low, responses (partial or complete) or prolonged stabilities were observed.

Dr Cruz *et al* from the Baylor College devised a strategy to decrease the risk of GvHD following donor lymphocyte infusion (DLI) in the context of allotransplant for B cell malignancies (leukaemias or lymphomas), but maintaining the beneficial antitumour and anti-viral effects of DLI. Donor lymphocytes were expanded by culture with antigen presenting cells engineered to present cytomegalovirus (CMV), adenovirus (Adv), and Epstein Barr virus (EBV) antigens, thus generating allogeneic virus specific T cells (VSTs). This were then transduced with an anti-CD19 CAR and infused in nine patients post-HSCT, either pre- or post-relapse. 2/6 relapsed patients showed signs of response, and those in remission remained relapse-free for the duration of the study. In two patients with EBV reactivation, donor cells expanded and decreased viral load.

Most studies to date have utilised either retro- or lenti-virus based vectors to transduce T cells. Albeit improvements in virus design and manufacturing have dramatically reduced safety issues, Dr Kebriaei *et al* from MD Anderson demonstrated the feasibility in the clinical setting of a completely non-virus-based method, designing a ‘sleeping beauty’ transposition-based vector, which yielded very encouraging results.

Finally, Hermanson *et al* from Minneapolis assessed the possibility to obtain effector cells *in vitro* from human induced pluripotent stem cells (iPSC) or human embryonic stem cells (hESC). They devised a good manufacturing practice-compatible, sleeping beauty-based protocol, to transduce iPSCs or hESCs with anti-CD19 or anti-mesothelin (to target mesotheliomas) and generate NK cells, which can kill target cells in a HLA non-restricted manner and without prior sensitisation. Their approach demonstrated effectiveness against ALL and mesothelioma cell lines.

The session moderators (Stephan Grupp from Philadelphia and Elizabeth Shpall from MD Anderson) highlighted a number of outstanding issues for translation of CARs in routine clinical practice: controlling the persistence of CAR-reprogrammed cells in the host, the optimal transducing vector, the design of the targeting construct (CD28 domain-containing second generation versus CD28 + OX40-domain containing third generation), problems associated with bulky disease, the optimal pre- and post-infusion therapy, what cell type is the best effector (T cells versus NK cells versus other immune cells), what disease is going to experience the highest impact.

In summary, although several important issues remain to be determined, CAR technology is now mature for transition into more widespread clinical use. It remains a technically demanding approach that can be employed only in dedicated centres for precise indications, such as to consolidate minimal residual disease or to produce prolonged remissions in refractory patients as a bridge to transplant. As highlighted above, most clinical studies to date focused on perfecting technical aspects of cell transduction, whereas research on alternative target specificities other than the B cell marker CD19 and few others is still limited to the pre-clinical setting, most likely due to the ethical and administrative constraints. Thus, it does not appear too far-fetched to imagine that the true potential of CARs still remains to be fully unveiled.

### Drug discovery and development

Several talks from high profile translational scientists dealt with the topic of designing drugs against novel targets, by taking advantage of recent genomics studies or other alternative approaches. Michael Deininger from Salt Lake City offered a panoramic view of the (sometimes unfulfilled) promises of tyrosine kinase inhibitors (TKIs), highlighting how the effectiveness of these drugs is not always consequential to their pharmacological activity in terms of biochemical inhibition and target specificity. Imatinib has had enormous clinical impact despite its relatively poor specificity, a problem partially overcome by second (dasatinib, nilotinib, and bosutinib) and third (ponatinib) generation BCR-ABL inhibitors. The reason for this success is due to the fact that in CML a single genetic lesion is the main disease driver and normal differentiation is maintained. However, in other hematological malignancies such as FLT3-mutated AML or JAK2-associated myeloproliferative neoplasms, extremely powerful and specific kinase inhibitors (quizartinib or ruxolitinib) have not had a similar impact, probably because in neither case the targeted mutation is in the founding leukaemia clone. On the other hand ibrutinib, which targets Bruton’s Kinase, a non-mutated mediator of constitutive B cell receptor signalling in CLL, appears to produce significant responses in CLL, albeit non-durable.

Julian Downward from Cancer Research UK addressed the long-standing issue of targeting mutated RAS, which despite being one of the most common and best studied cancer drivers, has to date resisted all attempts of drug design because of its biochemical idiosyncrasies. Dr Downward suggested three ‘collateral’ approaches to circumvent these problems: targeting the interaction between RAS and PIK3CA, which is crucial for mutated RAS activity even in the presence of wild type PIK3CA [6]; targeting the IGF1 pathway, to which RAS-mutated cells appear to be selectively addicted [7], and targeting downstream effectors of mutated RAS, like the transcription factor GATA2 [8].

Craig Thompson from the MSKCC showed very interesting and surprising results on the efficacy of IDH inhibitors on preclinical models of IDH-mutated AML. The drug, developed by Agios pharmaceuticals, was recently published to have *in vitro* efficacy against primary AML cells [9]. Thompson’s group studied a mouse model of IDH-mutated leukaemia; initially, they witnessed a seemingly paradoxical increase in peripheral white blood cells; subsequently, this was found to be a massive wave of differentiation of leukaemia blasts, which declined to undetectable levels in what appeared as a complete remission. This response is reminiscent of the differentiation induced by ATRA in APL and is going to be translated to clinical studies soon.

Dr Guy Sauvageau from Montreal showed the initial results of their cleverly designed drug screen, aimed at finding drugs able to eradicate leukaemia stem cells (LSCs) based on their underlying mutations. LSCs have been difficult to screen because of their propensity to undergo differentiation or death after brief *in vitro* culture. Thus, the authors first searched for a pathway able to maintain LSCs in culture and identified the aryl hydrocarbon receptor (AHR) as an essential target. Using AHR inhibitors, they were able to maintain LSCs in defined culture conditions for prolonged times, and performed several screens with compound libraries on 21 primary leukaemias representative of most biological AML classes, with an extremely high hit identification efficiency of 27%. Although he did not reveal the identity of the hits, he showed that specific leukaemias are sensitive to specific sets of compounds, such as steroids or TKIs, and that drug response is able to retrospectively prognosticate very well the outcome of patients.

### Non-coding RNA and epigenetics

Inspiring talks were proposed by several high profile researchers (Kristian Helin from Copenhagen, Rick Young from MIT, Howard Chang from Stanford, Andy Feinberg from Johns Hopkins, PP Pandolfi from Harvard, and John Stomatoyannopoulos from Seattle) on the fascinating topics of gene regulation through epigenetic and non-coding RNA-mediated mechanisms, and on the increasingly evident role their deregulation plays in the genesis of leukaemia, lymphoma and other tumours. Here, I will provide a synthetic account without reference to the single talks for the sake of clarity. According to estimates based on the recently published ENCODE consortium studies, and contrary to a dogma that appeared established until a short while ago, the total fraction of the human genome transcribed in the various cells that make up an individual body is well above the mere 2% that constitutes the protein-coding exome, averaging instead between 60% and 90%. The vast majority of these transcripts are made up of non-coding RNAs of sizes ranging between few hundred base pairs, like microRNAs, and several thousand base pairs, called long non-coding RNAs (lncRNA) [10]. For some, a regulatory function is now well understood, but the overwhelming majority is in effect completely uncharacterised. The study of non-coding RNAs is still in its infancy, for they are intrinsically more difficult to study than ‘traditional’ messenger RNAs: the language regulating their activity is unknown, thus the functional consequences of mutations in their sequence are hard to predict and to exploit for genotype-phenotype studies. Nevertheless, roles for miRNAs and, more recently, for lncRNAs such as HOTAIR in breast cancer or PRNCR1 and PCGEM1 in prostate cancer, have been elucidated [11].

Much better established is the role played by disruption of chromatin regulation in leukaemia and lymphoma, in particular histone methylation by components of the MLL and polycomb complexes and DNA methylation/demethylation by DNA methyltransferases and the recently characterised TET proteins [12]. More on this topic will be found below in the context of disease-specific pathobiology.

## Disease specific pathobiology

### Myeloid malignancies

Arguably one of the most important discoveries communicated at this year’s ASH was in the field of myeloproliferative neoplams (MPN), where an unresolved issue was the identity of mutated genes in the cases in which JAK2, the most commonly mutated gene, is wild type [13]. Two groups independently came to the same finding, one headed by Tony Green in Cambridge (Jyoti *et al*) and the other by Robert Kralovics in Vienna (Klampfl *et al*): the protein chaperone calreticulin is the culprit, being mutated in the majority (70%–90%) of patients with wild-type JAK2 MPNs, in particular essential thrombocythemia and primary myelofibrosis (but not polycythemia vera). This provides an important piece in the puzzle of this phenotypically heterogeneous but, as we now see, genetically homogeneous group of diseases. Almost all mutations occur in exon 9 and result in a frameshift that cause loss of the C-terminus and alters the protein’s function in a dominant-negative way. This finding will probably lead to the development of new laboratory tests for the rapid diagnosis of MPN and its differential from non-malignant myeloproliferation.

In the AML field, several papers investigated the functional aberrations caused by mutated genes of more recent identification. Three studies by Tim Ley’s laboratory in St Louis showed intriguing findings on the DNMT3A DNA methyltransferase, which is mutated in >30% of normal karyotype AMLs and correlates with poor prognosis [14]. David Russler-Germain showed that the most common mutation, R882H, causes a dominant loss of methyltransferase activity, thus explaining why these mutations almost invariably occur in heterozygosity. The *in vivo* effect was demonstrated by generating a doxycycline-inducible DNMT3A-R882H mouse (presented by Angela Verdoni), which showed a haematopoietic phenotype characterised by differentiation block, anaemia and peripheral blasts (though no overt AML) with incomplete penetrance. Intriguingly, these animals completely and reversibly lost their hair upon DNMT3A-R882H induction, suggesting an important role for DNMT3A in epithelial homeostasis too. Finally, Cristopher Cole showed that wild-type DNMT3A is required for PML/RARA-dependent leukemogenesis in a mouse model of APL, thus explaining why PML/RARA and DNMT3A are mutually exclusive.

A new kid on the block of myeloid neoplasm-associated genes is the cohesin complex, involved in genome stability, and gene expression regulation. Its components RAD21, SMC3, and SMC5 were previously found to be mutated in about 10%–15% of adult AMLs and in MDS [15].

Norio Shiba from Gunma University in Japan showed that these mutations are also found in paediatric AMLs and are associated with a relatively favourable outcome. On the other hand, Felicitas Thol from Hannover found that in adult patients cohesin mutations were not associated with significant outcome differences. The prevalence of cohesin mutations seemed to be higher in AMLs secondary to MDS than in primary MDS patients (Makishima *et al*, Cleveland). Time will tell if this interesting group of genes plays a role in risk stratification and drug development.

### Lymphoid malignancies

Jonathan Reichel and colleagues performed the first whole exome sequencing (WES) in Hodgkin’s lymphoma, an endeavour made particularly difficult by the scarcity of the disease-specific Reed–Sternberg (RS) cells in clinical samples. The authors managed to increase the yield and purity of the samples by isolating RS cells by multicolour flow cytometry. They identified recurrent mutations or copy number alterations involving genes important for antigen presentation (B2M, altered in 80% of the cases), NF-ĸB activation (A20/TNFAIP3), chromosome integrity (BCL7A), and protein ubiquitination (HECW2 and UBE2A). B2M alterations led to a lack of expression of MHC class I protein complex on the cell surface. Interestingly, B2M was never altered in cases histologically classified as ‘mixed cellularity’, possibly providing a sound diagnostic biomarker.

Mamiko Sakata–Yanagimoto from Tsukuba identified a novel recurrent mutation in *RHOA* (c.G50T/p.G17V) in angioimmunoblastic lymphoma by a WES approach, which they subsequently found mutated in about 70% of cases in a bigger validation cohort, together with mutations in usual suspects such as TET2, IDH2, and DNMT3A.

Davide Rossi and colleagues from Novara in Italy addressed the interesting issue of low-allele frequency p53 mutations and their prognostic impact in CLL (also discussed in AML, see above). By employing targeted deep sequencing, they were able to identify Sanger-undetectable p53 mutations in 9% of CLL patients. Sometimes this was the only identifiable genetic abnormality. Importantly, patients with subclonal p53 mutations had a twofold higher risk of death (*p* = .023), demonstrating that what lies beneath the limit of detection of conventional techniques is of extreme clinical importance.

Wendy Beguelin from Weil Cornell in New York shed some light on the intriguing mechanism of lymphomagenesis exerted by the mutated histone methyltransferase EZH2, a member of the polycomb complex [16]. Mice with a B cell-specific loss of EZH2 or treated with an EZH2 inhibitor completely failed to form germinal centres, whereas mice engineered to express the lymphoma-associated EZH2Y641F mutant exhibited massive germ cell hyperplasia. The authors elegantly showed that a crucial set of genes is overexpressed and lacks histone methylation (on histone 3 lysine 27) in the absence of EZH2, and is repressed and undermethylated when EZH2 is mutated. This same set of genes is silenced in human diffuse large B cell lymohoma (DLBCL). The authors found that EZH2 and the well known germinal centre regulator BCL6 cooperate in controlling the expression of this gene set and demonstrate that compound inhibition of EZH2 and BCL6 has significant activity both *in vitro* and on primary xenograft models of DLBCL, suggesting a promising path for developing a combinatorial drug approach.

Jan Kroncke from Ben Ebert’s group in Boston shed some important light on the poorly understood mechanism of action of lenalidomide in multiple myeloma. He and colleagues found that the IKZF1 and IKZF3 transcription factors are selectively targeted for proteasomal degradation by lenalidomide-dependent ubiquitin ligase activity. A 58-aminoacid domain on IKZF1 was found to be essential for degradation and a specific mutation in a critical residue rendered cells lenalidomide resistant. This explains the mechanism of action of this drug, since IKZF1 and IKZF3 are essential selectively for the survival of myeloma and other T cell malignancies [17].

## Clinical research

### Myeloid malignancies

As mentioned above, comparatively few significant treatment advancements were established this year, especially in the myeloid field.

Several papers discussed the addition of the anti-CD33 antibody gemtuzumab ozogamicin (GO) to standard induction chemotherapy, a matter of heated debate in the recent past [18]. Robert Hills, on behalf of the GO trialists collaborative group, presented a meta-analysis of the five randomised trials published so far, possibly clarifying the indications for GO use. With little heterogeneity among studies, GO showed improvement in OS and PFS in low and intermediate risk AMLs, with little benefit in high risk patients. No effect was observed on remission rate, and the slight increase in early mortality initially observed in the SWOG0106 (which led to GO withdrawal from the market) was not statistically significant in the meta-analysis and appeared to correlate with high GO dosage. Thus, careful patient selection is crucial for optimal GO usage, a point further strengthened by a 25-year retrospective analysis of outcome determinants in CBFb-mutated AML (a favourable risk group) in the UK, presented by Alan Burnett. After multivariate analysis, the introduction of GO significantly improved OS, as well as the introduction of high dosa Ara-C consolidation. Finally, Dr Pollard from Seattle showed that the negative prognostic impact of CD33 overexpression was abrogated when GO was used in the COG AAML0531 trial, further suggesting that GO is effective in a biologically defined and predictable subset of patients.

Several studies reported on the use of kinase-inhibiting small molecules in both AML and myeloproliferative disorders. Guido Marcucci from Ohio reported on the initial results of the CALGB 10801 (Alliance) multicentre study, and showed non-inferiority in terms of toxicity and remission rates of adding the KIT inhibitor dasatinib to standard induction and consolidation therapy for CBFb mutated AML. Dr Cortes from MD Anderson preliminarily showed that in FLT3ITD AML the dose of toxic but highly effective FLT3 inhibitor quizartinib [19] can be reduced with no loss in efficacy while improving toxicity. Dr Harrison from London reported the planned interim analysis of the Sanofi-sponsored JAKARTA-2 study investigating the second generation JAK2-inhibitor Fedratinib in myelofibrosis/polycythemia patients previously treated with ruxolitinib. The majority of the 27 patients in study experienced clinical benefit through reduced splenomegaly and symptom burden, with manageable toxicity.

Results from early phase trials with non-kinase targeting biological therapy were also presented. The SGI-110, a novel subcutaneous DNAhypomethylating agent, showed efficacy and tolerability in relapsed/refractory or chemotherapy-unfit AML patients (Kanatarjian *et al*). The p53-stabilising MDM2 antagonist RG7112 in combination with Ara-C showed tolerability and a favourable biomarker profile in a phase I trial (Yee *et al*).

Dr Burnett tried to put the final word on a debated issue by presenting data from the NCRI AML16 trial, which investigated whether etoposide or ATRA provide any benefit when added on top of a Daunorubicin + Ara-C regimen in non-M3 AML. No difference was observed for either drug, even in the NPM-mutated/FLT3ITD subgroup in which ATRA showed benefit in a previous study.

Given the extreme biological heterogeneity of AML, criteria for risk stratification have been intensely investigated. Dr Walter from Seattle retrospectively analysed data for 4550 non-M3 AMLs from the UK, Europe and the USA and disappointingly found that the ability of routinely available clinical and molecular variables to predict refractoriness to induction or early relapse is limited, with AUCs in the order of 0.70–0.75. Thus, the need for new biomarkers remains compelling.

### Lymphoid malignancies

The role of lenalidomide was consolidated in myeloma and ventured into the lymphoma field with studies from Weill Cornell in New York (Ruan *et al*), UC Davis (Yamshon *et al*), and Philadelphia (Chon *et al*), which reported on the activity of chemotherapy-free regimens based on lenalidomide + rituximab in indolent NHL or mantle cell lymphoma, with high response rates in the order of 70%, even in previously rituximab-refractory patients. Preliminary results from several phase II trials on PI3K inhibitors on relapsed/refractory NHL were presented by authors from Washington University in St Louis, the Dana Farber Cancer Centre and Munich, all showing promising results with response rates in the order of 50% and acceptable safety profiles.

Regarding multiple myeloma, particularly interesting was the practice-changing study by Facon *et al*, presented in the plenary session, showing initial results of the ‘FIRST’ phase III trial of lenalidomide + low-dose dexamethasone versus standard melphalan, prednisone, and thalidomide (MPT) in newly diagnosed multiple myeloma patients ineligible for transplantation. At 37 months of median follow-up, there was a 28% reduction in risk of progression (HR = 0.72; *p* = 0.00006) and a 22% reduction in risk of death (HR = 0.78, *p* = 0.01685). The study was particularly praised for its vicinity to real clinical practice, as the authors managed to recruit a large cohort (*n* = 1623) and included patients with kidney failure, which are normally excluded from late phase trials.

In a similar vein, the Roche-sponsored multicentre CLL11 trial (Goede *et al*) evaluated the second-generation glycoengineered anti-CD20 antibody obinutuzumab (GA101) [21] plus chlorambucil (G-Clb) versus rituximab plus chlorambucil (R-Clb) versus chlorambucil alone (Clb) in CLL, allowing enrolment of patients with multiple comorbidities including kidney failure. After 23 months of median follow-up, the G-Clb was better than R-Clb and Clb in terms of PFS (26.7, 16.3, and 11.1 months, respectively). OS analysis demonstrated a benefit of G-Clb over Clb (HR 0.41, CI 0.23-0.74, *p* = 0.002) and a trend approaching significance for R-Clb over Clb (HR 0.66, CI 0.39-1.11, *p* = 0.113) [20].

Also of interest in the CLL field was the late-breaking abstractpresented by Furman *et al*: a phase three study comparing the oral PI3Kδ inhibitor idelalisib [22] + rituximab versus placebo + rituximab for previously treated, progressing CLL patients. Comorbidities were allowed up to a cumulative score of six, but renal dysfunction was an exclusion criteria. The study was stopped at the planned interim analysis, consisting of >50% of the planned events, when the experimental arm showed superiority in terms of PFS (median not reached versus 5.5 months in the control arm), PFS rate at 24 weeks (93% versus 46%), response rate (81% versus 13%; odds ratio 29.9; *p* = 3.0 × 10 19) and OS (HR = 0.28, 95% CI = 0.09–0.86, *p* = 0.018), with an acceptable safety profile.

The Bruton kinase inhibitor ibrutinib was, as could be expected, the centre of much attention. Susan O’Brien from MD Anderson presented an update of their single-centre trial testing the combination of ibrutinib and rituximab in high risk-CLL. After 14 months of median follow-up, the high response rate (95%) appeared long-lasting, with improvements in quality of life and a good toxicity profile, although some patients came off study due to high grade toxicity which could not be unequivocally assigned to the experimental drug [23].

Finally, Treon *et al* presented very interesting data on the efficacy of ibrutinib in patients with relapsed/refractory Waldenstrom’s macroglobulinemia (WM). The rationale of this approach lies in the recent discovery of mutations in the signal transducers MYD88 (which mediates Toll-like receptor signalling) and CXCR4 in a majority of WM patients. Both these mutations were shown to activate the Bruton kinase *in vitro*, and to sensitise cells to ibrutinib. Of the 63 enrolled patients, 34 had their bone marrow reassessed after six months and showed a decrease from 70% to 45% in disease involvement for these patients (*p* = 0.0006). Response was correlated with the presence of CXCR4 mutation but not with MYD88.
